# Administration of *Lactobacillus salivarius* LI01 or *Pediococcus pentosaceus* LI05 prevents CCl_4_-induced liver cirrhosis by protecting the intestinal barrier in rats

**DOI:** 10.1038/s41598-017-07091-1

**Published:** 2017-07-31

**Authors:** Ding Shi, Longxian Lv, Daiqiong Fang, Wenrui Wu, Chenxia Hu, Lichen Xu, Yanfei Chen, Jing Guo, Xinjun Hu, Ang Li, Feifei Guo, Jianzhong Ye, Yating Li, Dewi Andayani, Lanjuan Li

**Affiliations:** State Key Laboratory for Diagnosis and Treatment of Infectious Diseases, Collaborative Innovation Centre for Diagnosis and Treatment of Infectious Diseases, the First Affiliated Hospital, College of Medicine, Zhejiang University, 310003 Hangzhou, China

## Abstract

Alterations in the gut microbiome have been reported in liver cirrhosis, and probiotic interventions are considered a potential treatment strategy. This study aimed to evaluate the effects and mechanisms of *Lactobacillus salivarius* LI01, *Pediococcus pentosaceus* LI05, *Lactobacillus rhamnosus* GG, *Clostridium butyricum* MIYAIRI and *Bacillus licheniformis* Zhengchangsheng on CCl_4_-induced cirrhotic rats. Only administration of LI01 or LI05 prevented liver fibrosis and down-regulated the hepatic expression of profibrogenic genes. Serum endotoxins, bacterial translocations (BTs), and destruction of intestinal mucosal ultrastructure were reduced in rats treated with LI01 or LI05, indicating maintenance of the gut barrier as a mechanism; this was further confirmed by the reduction of not only hepatic inflammatory cytokines, such as TNF-α, IL-6, and IL-17A, but also hepatic TLR2, TLR4, TLR5 and TLR9. Metagenomic sequencing of 16S rRNA gene showed an increase in potential beneficial bacteria, such as *Elusimicrobium* and *Prevotella*, and a decrease in pathogenic bacteria, such as *Escherichia*. These alterations in gut microbiome were correlated with profibrogenic genes, gut barrier markers and inflammatory cytokines. In conclusion, *L*. *salivarius* LI01 and *P*. *pentosaceus* LI05 attenuated liver fibrosis by protecting the intestinal barrier and promoting microbiome health. These results suggest novel strategies for the prevention of liver cirrhosis.

## Introduction

Liver cirrhosis has a high risk of mortality because the normal liver architecture is disrupted, ultimately impairing liver functions. This condition is the 14th most common cause of death worldwide^1^ and has a poor prognosis. Liver cirrhosis is caused by various factors, including viral infection, schistosomiasis, alcoholism, cholestasis and metabolic disorders. However, the underlying molecular mechanisms of liver cirrhosis are still poorly understood, and novel treatment strategies are urgently needed.

Growing evidence has demonstrated that the gut microbiota are closely related to liver cirrhosis progression. Our previous study found that increased abundance of *Streptococcaceae* was positively correlated with Child-Turcotte-Pugh (CTP) score in patients with cirrhosis, whereas *Lachnospiraceae* decreased significantly and was negatively correlated with CTP score^2^. Using quantitative PCR, researchers previously showed that 16 S rRNA gene copy numbers for *Bifidobacteria*, *Lactobacillus*, *Pediococcus*, *Leuconostoc* and *Weissella* were substantially reduced in patients with decompensated HBV cirrhosis compared to those of healthy controls and asymptomatic HBV carriers. Moreover, the copy numbers and detection rate of virulence genes from *Escherichia coli* were significantly increased in decompensated HBV cirrhotic patients^3^. Several opportunistic pathogenic bacteria from the phyla *Firmicutes* and *Proteobacteria* were enriched in the gut of primary biliary cirrhosis (PBC) patients and may be critical for the onset or development of PBC by interacting with metabolism and immunity^4^. Another study demonstrated that oral commensals, such as *Campylobacter* and *Haemophilus parainfluenzae*, invade the gut in patients with liver cirrhosis. This phenomenon did not occur in healthy controls, suggesting that invasion via the oral route might play an important role in the progression of liver cirrhosis^5^. Using a specific functional gene array for analysing the human microbiome, researchers revealed variations in the functional genes of the gut microbiome, suggesting that cirrhosis may have distinct influences on the metabolic potential of gut microbial communities^6^. In cirrhotic patients with hepatic encephalopathy, altered specific gut microbial taxa, such as *Autochthonous* and *Enterobacteriaceae*, may influence neurons and astrocytes related to cirrhosis-associated brain dysfunction^7^. Therefore, prevention of or adjuvant treatment for liver cirrhosis through modulation of the gut microbiome composition to stabilize the intestinal flora balance has vital clinical significance.

This study first investigated the effects of five different probiotics, which were efficacious in other liver diseases, on CCl_4_-induced liver cirrhosis in rats. *Lactobacillus salivarius* LI01 and *Pediococcus pentosaceus* LI05 demonstrated significant protective effects against liver injury and BT in our previous study^8^. *Clostridium butyricum* MIYAIRI 588 improved non-alcoholic fatty liver disease (NAFLD) by decreasing accumulation of lipid droplets in the liver and regulating cholesterol catabolism enzymes and excretion transporters^9^. Pepper powder fermented by *Bacillus licheniformis* SK1230 may have a beneficial effect on NAFLD by inhibiting fat accumulation and improving lipid metabolism in mice fed a high-fat diet^10^. The *Lactobacillus rhamnosus* GG strain reduced gut leakiness and attenuated oxidative stress and inflammation on a rat model of alcoholic steatohepatitis^11^. Furthermore, we performed a comprehensive analysis of the gut flora, intestinal barrier, microbial molecular receptors, and inflammatory pathways to elucidate the mechanisms underlying the effects of specific probiotics on liver cirrhosis.

## Materials and Methods

### Strains and culture conditions

We selected the five following strains in this study: *Lactobacillus salivarius* LI01 (CGMCC 7045), *Pediococcus pentosaceus* LI05 (CGMCC 7049), *Clostridium butyricum* MIYAIRI 588®, *Bacillus licheniformis* Zhengchangsheng® and *Lactobacillus rhamnosus* GG (ATCC 53103). *L*. *salivarius* LI01 and *P*. *pentosaceus* LI05 were originally isolated from healthy volunteers. *C*. *butyricum* MIYAIRI 588® was isolated and cultured by the Miyarisan Pharmaceutical Co., Ltd. (Tokyo, Japan). *B*. *licheniformis* Zhengchangsheng® (CMCC 63516) was isolated and cultured by the Northeast Pharmaceutical Group Co., Ltd. (Shenyang, China). All strains were cultured in MRS broth (Difco, BD, Sparks, MD, USA) for 24 h at 37 °C. The *B*. *licheniformis* Zhengchangsheng strain was incubated in an aerobic atmosphere, and the other strains were cultured anaerobically. Cells were obtained by centrifugation at 8,000 × g for 10 min at 4 °C. Subsequently, the cells were washed twice with physiological saline at 4 °C and resuspended to a concentration of 3 × 10^9^ colony-forming units (CFUs)/ml in physiological saline prior to use.

### Experimental design and model of cirrhosis

**Figure 1:**
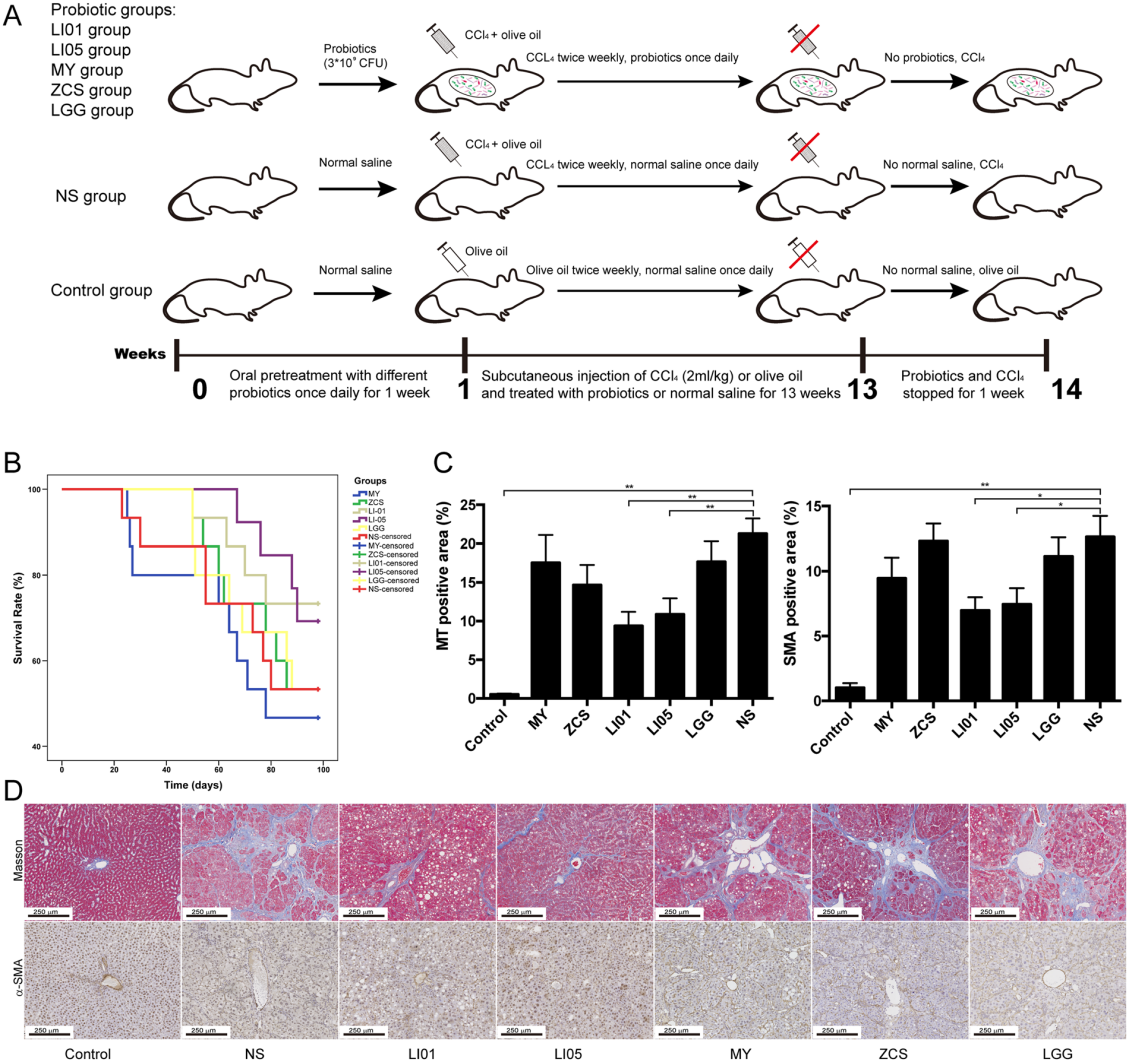
Treatment with *L. salivarius* LI01 or *P. pentosaceus* LI05 prolonged survival and attenuated liver fibrosis. (**A**) Experimental protocol during 14 weeks. LI01 group, cirrhotic rats treated with *L. salivarius* LI01; LI05 group, cirrhotic rats treated with *P. pentosaceus* LI05; MY group, cirrhotic rats treated with *C. butyricum*; ZCS group, cirrhotic rats treated with *B. licheniformis*; LGG group, cirrhotic rats treated with *L. rhamnosus* GG; NS group, cirrhotic rats treated with normal saline. (**B**) Survival times of the Control, NS, LI01, LI05, MY, ZCS, and LGG groups. LI01 group vs. NS group (P < 0.1), LI05 vs. NS group (P < 0.05). (**C**) Positive α-SMA or MT staining was analysed with Image-Pro Plus 6.0 software. (**D**) Representative images of liver histology and fibrillar collagen deposition assessed by MT staining. Activated HSCs evaluated by immunohistochemical staining of α-SMA from the Control, NS, LI01, LI05, MY, ZCS, and LGG groups. Scale bar: 250 μm. All data are given as the mean ± SEM. ^*^P < 0.05 and ^**^P < 0.01 vs. normal saline-treated cirrhotic rats in the NS group.

We designed the following liver cirrhosis experimental protocol as shown in Fig. 1A. Male pathogen-free Sprague–Dawley rats weighing 250 to 350 g were randomly divided into 7 groups as follows: a healthy control group treated with normal saline (Control group; n = 8), a CCl4-induced cirrhosis group treated with normal saline (NS group; n = 15), and five CCl4-induced liver cirrhosis groups treated with *L. salivarius* LI01 (LI01 group; n = 15), *P. pentosaceus* LI05 (LI05 group; n = 15), *C. butyricum* (MY group; n = 15), *B. licheniformis* (ZCS group; n = 15) or *L. rhamnosus* GG (LGG group; n = 15). An orogastric tube was used to administer 1 ml of normal saline in the NS and Control groups or 1 ml (3 × 10^9^ CFU/ml) of bacterial cells freshly prepared as previously described^8^ (once daily for 13 weeks; pre-treatment with probiotics was initiated one week prior to the induction of cirrhosis).

Cirrhosis was induced via a subcutaneous injection of a 50% (V/V) CCl_4_ solution in olive oil (Sigma-Aldrich, St. Louis, MO) into the dorsal region twice per week at a dose of 2 ml/kg. The dose was administrated at the same time as 0.35 g/L phenobarbital (Sigma-Aldrich) in the rats’ drinking water. In the healthy control rats, CCl_4_ was replaced with olive oil. One week after the last CCl_4_ and probiotic dose, samples were collected for analysis. All procedures were performed according to the 2011 National Institutes of Health Guide for the care and use of laboratory animals. The study was approved by Animal Care and Use Committee of the First Affiliated Hospital, School of Medicine, Zhejiang University.

### Biochemical indicators of liver function

The serum levels of alanine aminotransferase (ALT), aspartate transaminase (AST), alkaline phosphatase (ALP), total bilirubin (TB) and gamma-glutamyl transpeptidease (GGT) were assessed by standard methods using a 7600–210 automatic analyser (Hitachi 7600–210; Hitachi, Tokyo, Japan).

### Endotoxin analysis

Blood was taken from the portal vein in pyrogen-free heparinized syringes during laparotomy. Venous blood samples (100 μL) were centrifuged at 3000 g for 10 min at room temperature to separate the serum for analysis. The endotoxin level was determined using a quantitative chromogenic Limulus amoebocyte lysate assay following the manufacturer’s instructions (Eihua Medical, Shanghai, China).

### Bacterial translocation and identification

Samples from the left lobe of the liver and mesenteric lymph nodes (MLN) were collected, weighed, and milled under aseptic conditions in glass homogenizers that had been autoclaved at 121 °C for 15 min. After appropriate dilution with physiological saline, the milled MLN samples and samples from arterial blood or portal blood were separately incubated under aerobic and anaerobic conditions on brain heart infusion agar (BHI, Oxoid, Thermo Fisher Biochemicals Ltd., Beijing, China) at 37 °C for 48 to 72 h.

### Histological examination

For histological evaluation, all liver samples and the terminal ileums were immediately fixed in 10% neutral buffered formalin at the time of death. Paraffin-embedded samples were cut into 4-μm-thick sections. Subsequently, the liver sections were stained with Masson’s trichrome (MT). Staining intensity was quantified by histomorphometry with Image-Pro Plus 6.0. Sections from the ileum were processed for H&E and Alcian blue staining and analysed by a pathologist who was blind to the groups. For scoring the degree of injury to the terminal ileum, the inflammatory cell infiltration (score, 0–4), goblet cell depletion (score, 0–4), mucosa thickening (score, 0–4), destruction of architecture (score, 0 or 3–4) and loss of crypts (score, 0 or 3–4) were also semi-quantitatively assessed^12^.

### Immunohistochemical staining and immunofluorescence

Liver sections (4 µm) were dewaxed, rehydrated regularly and treated with 3% H_2_O_2_. The specimens were incubated overnight at 4 °C with anti-𝛼-SMA (1:500, Invitrogen). The cryostat sections of the terminal ileums were incubated with anti-ZO-1 (1:100, Invitrogen) antibodies overnight at 4 °C, followed by incubation with FITC-conjugated goat anti-rabbit secondary antibody (1:100, Beyotime) for 1 h at room temperature. The sections were then washed in PBS, mounted with mounting medium (Beyotime) and photographed under a fluorescence microscope (Eclipse 80i; Nikon, Tokyo, Japan).

### Electron microscopy

Ileal mucosal specimens were collected from various groups fixed and embedded as described^13^. The ultrastructure of the intestinal mucosa was analysed using a Philips Tecnai 10 electron microscope (Philips, Eindhoven, The Netherlands).

### DNA and RNA extraction

DNA was extracted from the caecal contents using a Qiagen stool kit (Qiagen, Hilden, Germany) according to the manufacturer’s protocol. Total RNA was isolated from liver samples and intestinal segments using an RNeasy mini kit (Qiagen, Hilden, Germany) according to the manufacturer’s protocol. The integrity of the DNA and RNA was verified by ethidium bromide staining. DNA and RNA extracts were stored until use at −20 °C and −80 °C, respectively.

### Real-time RT-PCR

The relative mRNA expression was measured in triplicate via the comparative cycle threshold method using a 7500 real-time PCR system (Applied Biosystems). The primer sequences (Integrated DNA Technologies) are shown in Supplementary Table 1. We used the housekeeping gene β-actin as the internal standard.

### Sequencing and bioinformatics

DNA from the isolated caecal contents was used as a template for the amplification of the 16 S rRNA V3 and V4 hypervariable region using the fusion dual barcoded PCR primers F319 (5′-ACTCCTACGGGAGGCAGCAG-3′) and 806 R (5′-GGACTACHVGGGTWTCTA AT-3′). Duplicate PCR products were pooled in equimolar concentrations and then purified using the Qiagen PCR purification kit (Qiagen, Hilden, Germany). Sequencing was performed on an Illumina MiSeq Instrument (Illumina Inc., San Diego, CA) using the 300 bp paired-end protocol.

Overlapping paired-end reads from the original DNA fragments were merged using FLASH (version 1.2.10). Quality control was conducted using FastQC (version 0.10.1). The assembled sequences were clustered using the CD-hit-est-based clustering method^14^. PyNAST software (http:/qiime.org/pynast/) was used to analyse and calculate the numbers of sequences and operational taxonomic units (OTUs) for each sample. Next, the sequences were grouped into various OTUs using Felsenstein-corrected similarity matrices; the sequences within an OTU shared at least 97% similarity. The Ribosomal Database Project (RDP) classifier, which is available from the RDP website (http://rdp.cme.msu. edu/classifier/), was used to classify the 16 S rDNA into distinct taxonomic categories by aligning the sequences to a curated database of taxonomically annotated sequences^15^. All 16 S rDNA sequences were mapped to the RDP database using BLASTN to determine the taxonomic assignments. Sequences sharing greater than 97% identity were used to associate a group of OTUs to specific species, whereas those with less than 97% identity were considered novel reads. The microbial diversity in individual conjunctival samples was estimated using rarefaction analysis, the Shannon diversity index (SDI), and the Chao 1 index. Principal coordinate analysis (PCoA) was performed for each sample using OTUs and the ade4 package within the R program (R version 2.15, http://www.R-project.org).

### Statistical analysis

For results of biochemical assays and histology score, the Kolmogrov-Smironv test was used to check for normality. Statistical differences between groups were estimated by Kruskal-waills test followed by the Mann-Whitney U test. The frequency of bacterial translocation was compared using the chi-squared test. Analysis of Similarities (ANOSIM) was used to test for clustering of microbial communities using weighted and unweighted UniFrac distance matrices. Wilcoxon rank sum test combined with the Benjamini-Hochberg method was applied to compare bacteria taxa. Correlations between variables were computed using the Spearman rank correlation. The values were presented as mean ± SEM if normally distributed; otherwise the values were presented as median (25th and 75th). P < 0.05 was considered significant. The data were analysed using SPSS version 20.0 for Windows (SPSS Inc., Chicago, IL, USA).

## Results

### *L. salivarius* LI01 and *P. pentosaceus* LI05 prolonged the survival time and protected against weight loss of CCl_4_-induced liver cirrhosis

Morality during this study was 53% in the MY group, 47% in the ZCS group, 27% in the LI01 group, 31% in the LI05 group (two rats were suffocated to be removed the group), 47% in the LGG group, and 47% in the NS group (Fig. 1B). Compared with the rats in NS group, the LI05 groups showed significantly increased survival time (P < 0.05, 92.3 ± 3.0 vs. 78.5 ± 7.6 days). We also observed a trend of increased survival time with LI01 supplementation (P < 0.1, 89.1 ± 4.2 vs. 78.5 ± 7.6 days; Table 1). No significant differences were found between the other probiotic groups. Body weight was an important indicator of rat health status during CCl_4_ administration. Notably, the body weights in the LI01 group (414.3 ± 11.2 g) and the LI05 group (412.3 ± 7.4 g) were significantly higher than those in the NS group (362.2 ± 9.2 g; P = 0.003 and P = 0.001, respectively) at the 6th week before ascites formation (Table 1), whereas the other probiotic groups showed no differences.

**Table 1:** Liver enzymes, bilirubin levels, survival time, body weight and endotoxin in the experimental groups.

	Control	MY	ZCS	LI01	LI05	LGG	NS
ALT (U/L)	33.5 ± 1.2^**^	201.3 ± 18.6	177.0 ± 19.8	182.2 ± 8.8	154.3 ± 12.5^*^	174.6 ± 15.2	234.2 ± 25.1
AST (U/L)	69.5 ± 3.4^**^	402.1 ± 52.7	347.0 ± 70.8	325.4 ± 10.7	262.7 ± 28.1^*^	352.9 ± 36.9	373.8 ± 23.2
GGT (U/L)	0.9 ± 0.6^**^	13.7 ± 1.7	12.6 ± 4.0	12.0 ± 1.2^*^	9.5 ± 1.8^**^	14.0 ± 1.5	19.8 ± 2.9
ALP (U/L)	51.1 ± 4.0^**^	311.2 ± 40.9	257.5 ± 29.3	271.5 ± 15.2	250.0 ± 37.8	331.0 ± 37.8	314.1 ± 27.7
TB (µmol/L)	1.5 ± 0.2^**^	28.0 ± 10.8	23.8 ± 2.9	20.7 ± 2.8	14.8 ± 3.9	18.3 ± 2.6	28.1 ± 5.6
Survival time (d)	98 ± 0 ^**^	73.6 ± 7.3	83.7 ± 4.7	89.1 ± 4.2	92.3 ± 3.0^*^	82.8 ± 5.2	78.5 ± 7.6
Body weight (g)	452.6 ± 11.6^**^	393.3 ± 12.5	387.1 ± 11.4	414.3 ± 11.2^**^	412.3 ± 7.4^**^	380.3 ± 10.3	362.2 ± 9.2
Endotoxin (EU/mL)	0.4 ± 0.1^**^	0.9 ± 0.2	0.8 ± 0.3	0.5 ± 0.1^**^	0.5 ± 0.1^**^	0.9 ± 0.2	1.2 ± 0.1

Values are expressed as the mean ± SEM. ^*^P < 0.05, ^**^P < 0.01 compared with the NS group.

### *L. salivarius* LI01 and *P. pentosaceus* LI05 improved liver functions and ameliorated liver fibrosis

Liver function analysis (Table 1) revealed that administration of *P. pentosaceus* LI05 significantly relieved the increase in serum ALT, AST and GGT (P < 0.05 and P < 0.01, respectively). Treatment with *L. salivarius* LI01 slightly decreased ALT (P = 0.07) and significantly decreased GGT level compared with the rats in NS group (P < 0.01). Conversely, treatment with *C. butyricum*, *B. licheniformis* or *L. rhamnosus* GG did not have a significant effect. After continuous CCl_4_ administration, typical pathological features of liver fibrosis were confirmed. Evaluation of the collagen content by MT staining of cirrhotic rats revealed increased collagen around the portal triad, lobules surrounded by bundles of collagen fibres, and the presence of large fibrous septa (Fig. 1C,D). Conversely, liver fibrosis and collagen deposition were only ameliorated in the rats treated with *L. salivarius* LI01 or *P. pentosaceus* LI05.

### *L. salivarius* LI01 and *P. pentosaceus* LI05 inhibited hepatic stellate cell (HSC) activation and attenuated the hepatic inflammatory response

The excessive production of extracellular matrix (ECM) during the development and progression of liver fibrosis is predominantly due to HSCs. Using immunohistochemical staining of α-SMA, a specific marker of activated HSCs, we found that the α-SMA-positive areas were substantially reduced by administration of probiotic LI01 or LI05 (Fig. 1C,D), whereas the livers in other strain-treated groups exhibited no differences in α-SMA compared to those of the NS group.

**Figure 2:**
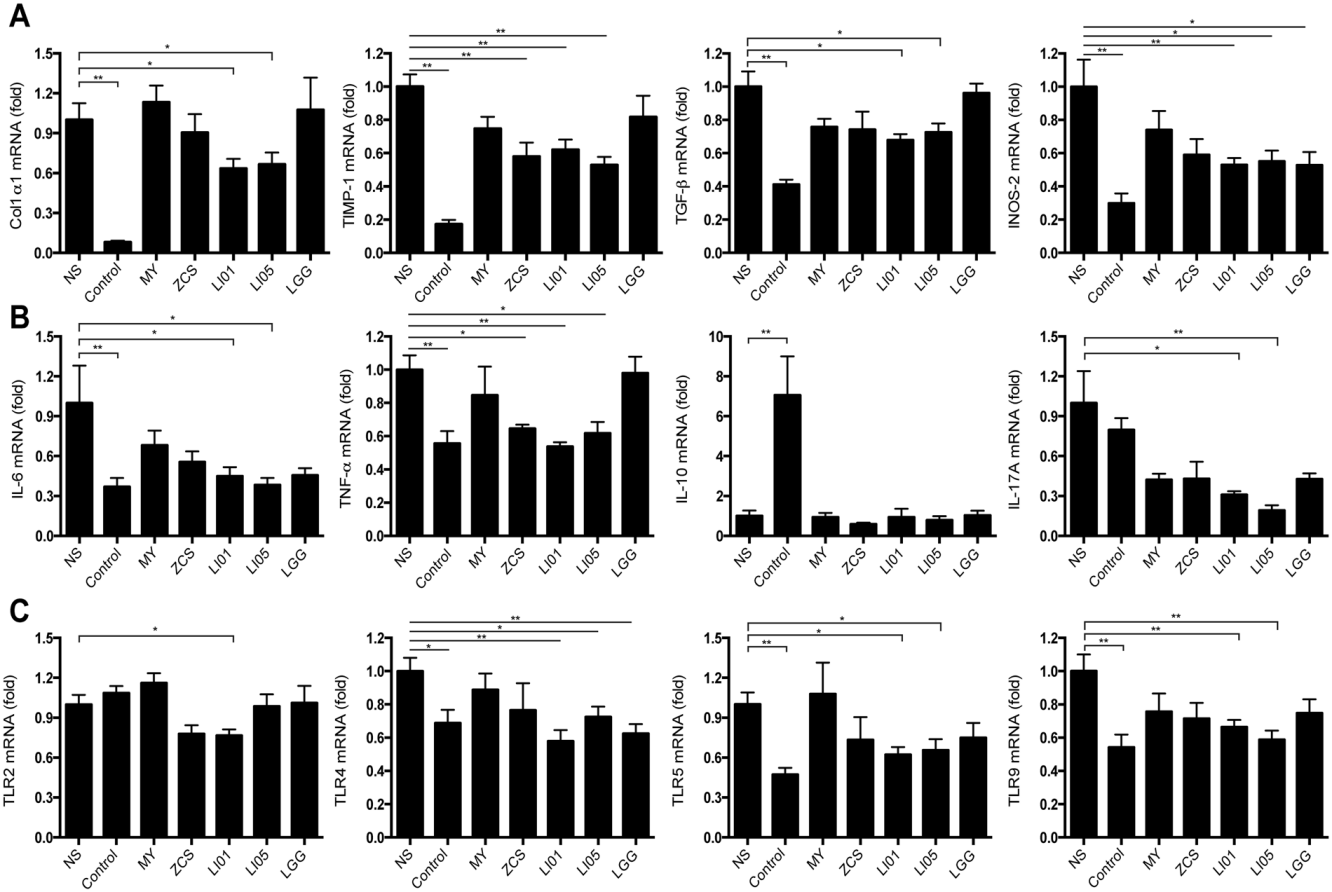
Treatment with *L. salivarius* LI01 or *P. pentosaceus* LI05 improved the inflammatory response and down-regulated profibrogenic genes and TLR family gene expression. (**A**) Hepatic expression of the profibrogenic genes Collagen α1, TIMP-1, TGF-β, and iNOS-2. (**B**) Hepatic expression of the inflammatory cytokines TNF-α, IL-6, IL-10 and IL-17A. (**C**) Hepatic expression of the TLR family members TLR2, TLR4, TLR5 and TLR9. Gene expression was determined by quantitative PCR analysis of the total mRNA extracted from liver fragments. Results are expressed as fold change relative to NS group. All data are given as the mean ± SEM. ^*^P < 0.05 and ^**^P < 0.01 vs. normal saline-treated cirrhotic rats in the NS group.

The following profibrogenic genes were significantly down-regulated in various groups compared with their expression in the NS group: Collagen α1 showed decrease in group LI01 and 1.5-fold decrease in group LI05; TIMP-1 showed 1.7-fold decrease in group ZCS, 1.6-fold decrease in group LI01 and 1.9-fold decrease in group LI05; TGF-β showed 1.5-fold decrease in group LI01 and 1.4-fold decrease in group LI05; and INOS-2 showed 1.9-fold decrease in group LI01, 1.8-fold decrease in group LI05 and 1.9-fold decrease in group LGG (Fig. 2A). Inflammatory cytokines play a key role in signal activation of liver fibrosis. As shown in Fig. 2B, IL-6 mRNA level showed 2.2-fold down-regulation in group LI01 and 2.6-fold down-regulation in group LI05 compared with its expression in cirrhotic rats from the NS group; TNF-α mRNA level showed 1.5-fold down-regulation in group ZCS, 1.9-fold down-regulation in group LI01 and 1.6-fold down-regulation in group LI05; IL-17A mRNA level showed 3.2-fold down-regulation in group LI01 and 5.2-fold down-regulation in group LI05; IL-10 expression was unchanged except in the Control group (7.1-fold up-regulated, P < 0.01). Notably, the administration of *C. butyricum* or *L. rhamnosus GG* did not significantly affect inflammatory cytokine regulation.

### *L. salivarius* LI01 and *P. pentosaceus* LI05 reduced plasma endotoxin levels and modulated TLR gene expression

Endotoxin is believed to be a key factor that contributes to the progression of liver damage. We tested the plasma endotoxin levels to evaluate the effect of the different probiotic strains on endotoxaemia (Table 1). Rats from the NS group (1.2 ± 0.1 EU/ml) showed significantly higher plasma endotoxin levels than those of rats from the Control group (0.4 ± 0.1 EU/ml, P < 0.01). A significant decrease in endotoxaemia was observed in the LI01 group (0.5 ± 0.1 EU/ml) and the LI05 group (0.5 ± 0.1 EU/ml) compared to that in the NS group (P < 0.01). There were no significant differences between the other probiotic groups and the NS group.

Endotoxin is an important ligand that activates specific Toll-like receptors (TLRs). Mechanistic investigations revealed that probiotic treatment strongly induced the expression of TLRs (Fig. 2C), which are involved in regulation of inflammation and bacterial infection. We observed significantly down-regulated expression of TLR2 in the LI01 group; TLR4 in all groups except MY and ZCS, and TLR5 and TLR9 in the LI01 and LI05 groups compared with the expression of these receptors in the cirrhotic rats in the NS group.

### *L. salivarius* LI01 and *P. pentosaceus* LI05 strongly reduced BT

As shown in Table 2, only treatment with *L. salivarius* LI01 decreased BT in all evaluated tissues compared with that of the cirrhotic rats treated with normal saline as follows: mesenteric lymph node (MLN) (4/11, P = 0.026), arterial blood (4/11, P = 0.026), portal blood (4/11, P = 0.026) and liver (3/11, P = 0.04). Moreover, treatment with *P. pentosaceus* LI05 decreased the BT incidence in the MLN (3/9, P = 0.024), arterial blood (3/9, P = 0.024) and portal blood (2/9, P = 0.007), treatment with *C. butyricum* decreased the BT incidence in the portal blood of the MY group (2/7, P = 0.02), and treatment with *L. rhamnosus* decreased the BT incidence in the arterial blood of rats in the LGG group (3/8, P = 0.039). However, *B. licheniformis* showed no significant effects on BT incidence.

**Table 2:** Incidence of bacterial translocation in the experimental groups.

Group	MLN	Arterial blood	Portal blood	Liver
MY	4/7 (57.1%)	4/7 (57.1%)	2/7 (28.5%)^*^	3/7 (42.9%)
ZCS	5/8 (62.5%)	5/8 (62.5%)	6/8 (75%)	5/8 (62.5%)
LI01	4/11 (36.3%)^*^	4/11 (36.3%)^*^	4/11 (36.3%)^*^	3/11 (27.3%)^*^
LI05	3/9 (33.3%)^*^	3/9 (33.3%)^*^	2/9 (22.2%)^**^	3/9 (33.3%)
LGG	4/8 (50%)	3/8 (37.5%)^*^	5/8 (62.5%)	3/8 (37.5%)
NS	7/8 (87.5%)	7/8 (87.5%)	7/8 (87.5%)	6/8 (75%)
Control	0/8 (0%)^**^	1/8 (12.5%)^**^	2/8 (25%)^*^	1/8 (12.5%)^*^

Results are shown as the number of rats with positive culture in different tissues; percentages are parenthesized. ^*^P < 0.05, ^**^P < 0.01 compared with the NS group. MLN, mesenteric lymph nodes.

### *L. salivarius* LI01 and *P. pentosaceus* LI05 improved the intestinal barrier integrity and protected the intestinal microstructure

**Figure 3:**
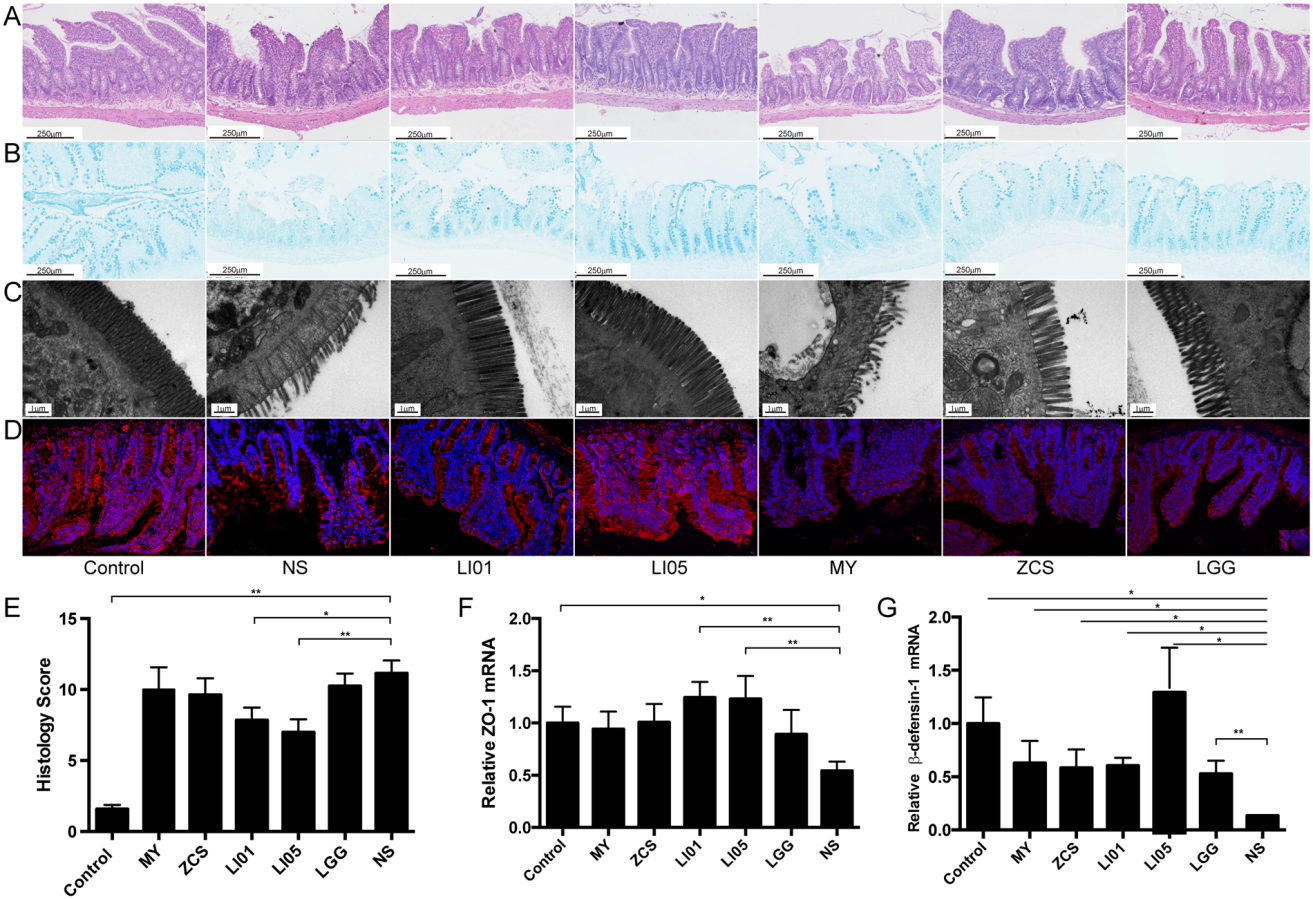
Treatment with *L. salivarius* LI01 or *P. pentosaceus* LI05 improved intestinal barrier function in cirrhotic rats. (**A**–**D**) Representative images of histological alternations in the ileum assessed by (**A**) H&E staining, (**B**) Alcian blue staining, (**C**) the ileal mucosal ultrastructure, and (**D**) ileal ZO-1 immunofluorescence (×20) from the Control, NS, LI01, LI05, MY, ZCS, and LGG groups. The scale bar in panel 1 is 250 μm and in panel 2 is 1 μm. (**E**) Ileum inflammation was monitored based on the histology scores following the criteria presented in the Materials and Methods. (**F**,**G**) Ileal ZO-1 and antimicrobial peptide β-defensin-1 gene expression were determined by quantitative PCR. All data are given as the mean ± SEM. ^*^P < 0.05 and ^**^P < 0.01 vs. normal saline-treated cirrhotic rats in the NS group.

We then focused on the intestinal barrier to explore the mechanism underlying the observed effect on BT. All cirrhotic groups demonstrated histological abnormalities of the terminal ileum; the level of inflammation was estimated in H&E- and Alcian blue-stained tissue sections of the terminal ileum (Fig. 3A,B), Although there were no differences in goblet cell depletion, mucosal thickening and crypts between each probiotic group, we observed *L. salivarius* LI01 or *P. pentosaceus* LI05 improved ileal inflammation marked by lower infiltration of leukocytes and more complete villi architecture compared to the rats in the NS group (Fig. 3E, Supplementary Table 2). Bacteria can penetrate the inner mucosal layer and contact the epithelium to elicit a beneficial or harmful effect. Therefore, we detected the ultrastructure of the ileal mucosa in the experimental groups to evaluate the microscopic changes in the intestinal mucosal integrity (Fig. 3C). The microvilli of the intestinal epithelial cells in the NS, MY, ZCS, and LGG groups were ruptured, sparse and stunted. However, the injured ultrastructure was substantially ameliorated in the LI01 and LI05 groups. Since the loosening of tight junctions is a major factor that contributes to pathological BT, we speculated that the probiotic strains LI01 and LI05 protected the intestinal barrier by enhancing these connections. To address this hypothesis, we measured ileal ZO-1 expression by quantitative PCR and immunofluorescence staining. In contrast with the other experimental groups, groups LI01 and LI05 showed higher levels of ZO-1 mRNA expression, which were verified by clear, uniform positive distributions of ZO-1 protein at the apical region of the intestinal epithelium (Fig. 3D,F). Notably, all probiotic strains could promote antimicrobial peptide β-defensin-1 secretion compared to the lowest mRNA level in the NS group (Fig. 3G).

### *L. salivarius* LI01 and *P. pentosaceus* LI05 increased the microbial richness and ameliorated microbiome dysbiosis

**Figure 4:**
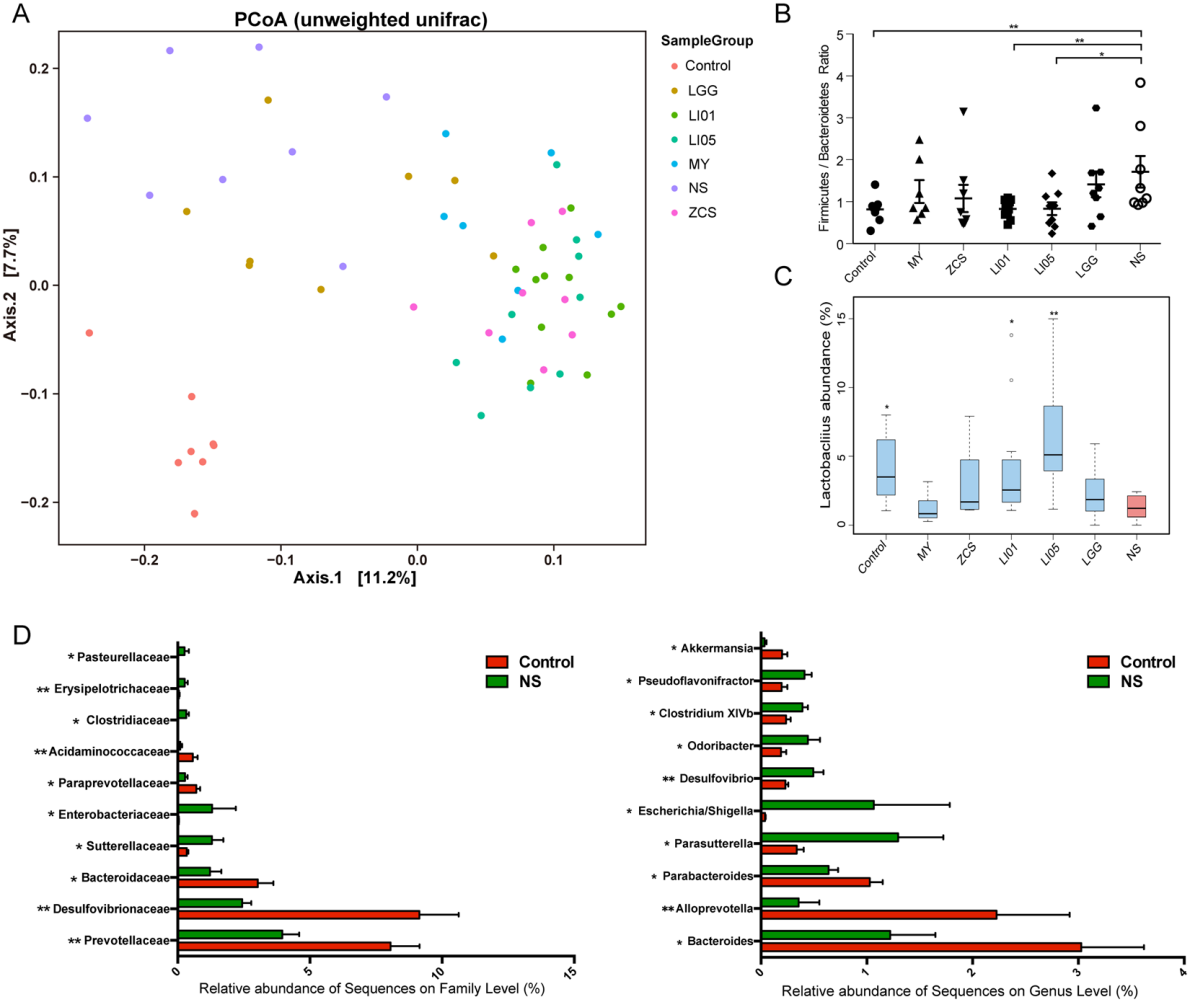
Comparison of the microbiome between different experimental groups. (**A**) A PCoA plot of the microbiota based on the results of the unweighted UniFrac metric. Each symbol represents a sample; the variance explained by the PCs is indicated on the axes (Unweighted PCoA; NS vs. Control, r = 0.82, P = 0.001; NS vs. LI01, r = 0.80, P = 0.001; NS vs. LI05, r = 0.81, P = 0.001). (**B**,**C**) The Firmicutes/Bacteroidetes (F/B) ratio and relative abundance of *Lactobacillus* in the Control, NS, LI01, LI05, MY, ZCS, and LGG groups. (**D**) Comparison of the ten most abundant taxa at the bacterial family and genus levels between the NS and Control groups. All data are given as the mean ± SEM. ^*^P < 0.05 and ^**^P < 0.01 vs. normal saline-treated cirrhotic rats in the NS group.

To gain further insights into the processes involved in BT reduction by probiotics, we investigated its impact on microbiota composition of caecal content by next-generation sequencing. The number of observed OTUs and the Chao1 index, the estimated microbial richness value, were significantly higher in the ZCS, LI01 and LI05 groups than those in the NS group (P < 0.01, Table S3). However, the overall microbial diversity was not significantly different between experimental groups based on the Shannon and Simpson indices (Table S3). Cirrhosis and probiotic treatment had a significant impact on the bacterial communities based on the PCoA plots (Fig. 4A, Figure S1). The unweighted and weighted UniFrac PCoA, which measured the phylogenetic similarities between microbial communities, showed a marked difference between the Control group and the NS group (Unweighted, P = 0.001, r = 0.82; Weighted, P = 0.002, r = 0.39; Table S4). Furthermore, weighted and Unweighted UniFrac analysis showed that the microbiota of the LI01 and LI05 group clustered separately from the NS group (ANOSIM, P < 0.01). In contrast, weighted metrics showed the microbiota of MY and LGG group had several intersection points with the NS group, indicating the closer microbial communities in these groups (ANOSIM, P > 0.05). On the other hand, the unweighted UniFrac analysis showed that the distinct microbial structure of the LI01 and LI05 group significantly separate from other probiotic groups (ANOSIM, P < 0.01, Table S4).

**Figure 5:**
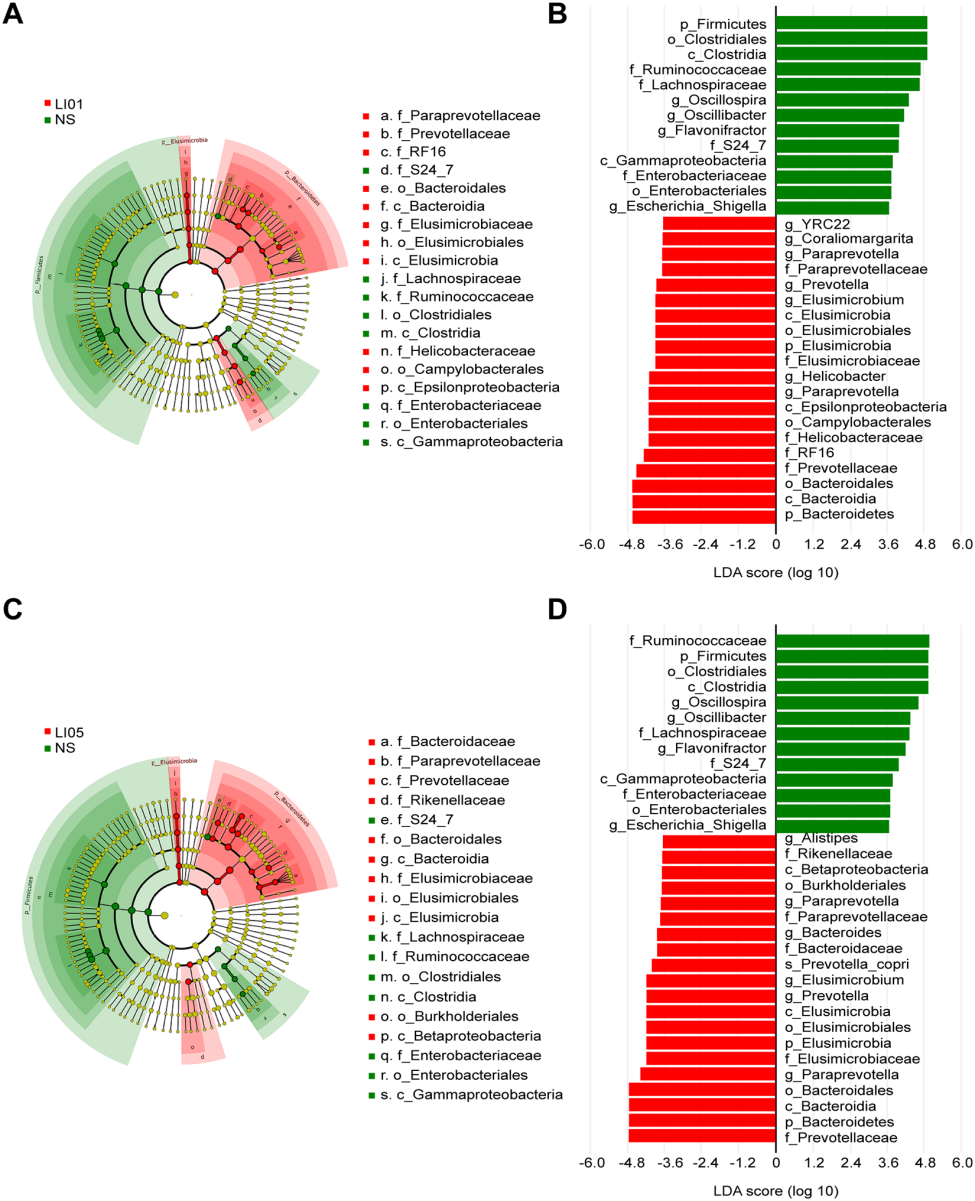
Treatment with *L. salivarius* LI01 or *P. pentosaceus* LI05 ameliorated microbiome dysbiosis in cirrhotic rats. (**A**–**D**) LEfSe prediction was used to identify the most differentially abundant taxa in the LI01 or LI05 group compared to the NS group. (**A**,**C**) The cladogram shows the phylogenetic relationship between the microbial taxa that were higher in probiotic samples compared to those in the cirrhotic rats in the NS group. (**B**,**D**) LDA scores showed significant bacterial differences between the NS and probiotic groups. Only the taxa meeting a significant LDA threshold value of >3.6 are shown. Red indicates the LI01 or LI05 group, and green indicates the NS group.

Despite the highly diverse bacterial communities and interindividual differences, cirrhosis clearly affected the intestinal microbial community. Compared with the controls, the rats with cirrhosis in the NS group demonstrated a marked increase in the Firmicutes/Bacteroidetes (F/B) ratio (1.7130 ± 0.3771 vs. 0.8183 ± 0.1113, P < 0.01). Additionally, only the probiotic strains LI01 (0.8287 ± 0.0630, P < 0.01) and LI05 (0.8329 ± 0.1512, P < 0.05) significantly decreased the ratio (Fig. 4B). Although *Lactobacillus* comprised a small proportion of microbial sequences, we found that its relative abundances were significantly higher in the LI01 and LI05 microbiome compared with those of the NS microbiome (Fig. 4C). The ten most abundant taxa that differed between the Control and NS microbiome at the family and genus levels are presented in Fig. 4D. Among these discriminatory taxa, 5 bacterial families and 4 genera were more abundant in the Control microbiome, and the other 8 microbial taxa were strongly enriched in the NS microbiome, including the families *Sutterellaceae*, *Enterobacteriaceae*, *Erysipelotrichaceae*, and *Pasteurellaceae* and the genera *Parasutterella, Escherichia*/*Shigella*, *Odoribacter*, *Pseudoflavonifractor*. Next, we used linear discriminant analysis effect size (LEfSe) to compare the estimated phylotypes of the probiotics and NS microbiome. LEfSe demonstrated that compared to the NS group, the phylum Bacteroidetes and the genera *Elusimicrobium*, *Paraprevotella*, and *Prevotella* were both enriched in LI01 and LI05 group, whereas the NS group was characterized by a preponderance of Firmicutes, *Enterobacteriaceae*, *Oscillospira*, *Oscillibacter*, *Flavonifractor* and *Escherichia*/*Shigella* (Fig. 5A–D).

### Correlations of gut bacteria with endotoxin, inflammatory cytokines, profibrogeneic genes, TLR and gut barrier marker indicating important roles in the regulation of liver cirrhosis

**Figure 6:**
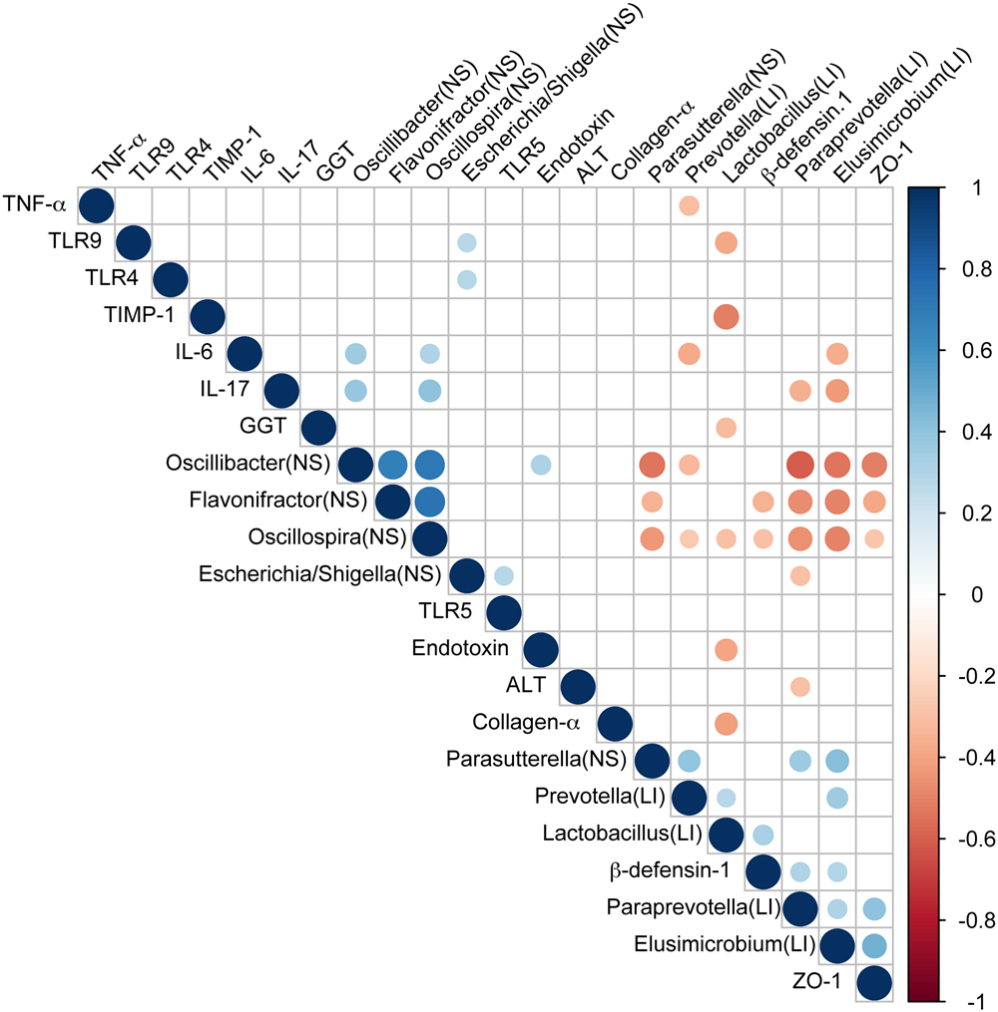
Gut microbiota changes are closely associated with the level of endotoxin, inflammatory cytokines, profibrogeneic genes and gut barrier marker. Heatmap of Spearman’s rank correlation cofficients of the relative abundances of alternation-related bacteria with inflammatory cytokines, profibrogeneic genes and gut barrier marker in all cirrhotic rats. The letters NS in parentheses on the heatmap indicates the microbiota was enriched in NS group; the letter LI in parentheses on the heatmap indicated the microbiota was enriched in LI01 and LI05 group. Those correlations with a coefficient r < 0.25 are deleted and not shown in this figure. Color key and circle size indicates the strength of correlation (r value). Dark blue indicates a more positive correlation; dark red indicates a more negative correlation; white indicates no correlation.

The relative abundance of several probiotics-altered gut bacteria were associated with endotoxin, inflammatory cytokines, profibrogeneic genes, TLR and gut barrier marker (Fig. 6). The altered gut microbiota in the NS group may be involved in the impairment of gut barrier and progression of liver cirrhosis. As a result, consistent negative linkages were detected between potentially pathogenic genera enriched in the NS group and beneficial ones enriched in probiotics groups, e.g. *Oscillospira* was negatively correlated with *Elusimicrobium* (r = −0.49, P = 0.00), *Lactobacillus* (r = −0.3, P = 0.02), and *Prevotella* (r = −0.27, P = 0.04). Liver and intestinal damage were highly associated with gut microbial genera. Among the various relationships, we found negative correlations between the gut barrier marker ZO-1 and *Oscillospira* (r = −0.28, P = 0.04), *Oscillibacter* (r = −0.5, P = 0.00), and *Flavonifractor* (r = −0.38, P = 0.00). Positive correlations were identified between ZO-1 and *Elusimicrobium* (r = 0.48, P = 0.00) and *Paraprevotella* (r = 0.4, P = 0.00). Specifically, *Elusimicrobium* was highly negatively correlated with IL-6 (r = −0.37, P = 0.00) and IL-17 (r = −0.43, P = 0.00), whereas *Oscillospira* was positively correlated with IL-6 (r = 0.3, P = 0.02) and IL-17 (r = 0.4, P = 0.00), indicating that *Elusimicrobium* and *Oscillospira* are strong opposite candidates for inflammatory cytokine regulation. Interestingly, the genus *Lactobacillus* was correlated negatively with the profibrogenic genes Collagen α1 (r = −0.4, P = 0.02) and TIMP-1 (r = −0.5, P = 0.00) and the liver function markers GGT (r = −0.32, P = 0.01) and endotoxin (r = −0.4, P = 0.00). In contrast, significant positive correlations were found between *Escherichia/Shigella* and TLR4 (r = 0.28, P = 0.03), TLR5 (r = 0.28, P = 0.03), and TLR9 (r = 0.27, P = 0.04).

## Discussion

The major finding in the present study was that long-term administration of *L. salivarius* LI01 or *P. pentosaceus* LI05 prolonged the survival time and substantially ameliorated CCl_4_-induced liver cirrhosis in rats. Compared with widely used probiotic strains (*L. rhamnosus* GG, *C. butyricum* MIYAIRI or *B. licheniformis* Zhengchangsheng), the probiotic strains LI01 and LI05 achieved this effect through several complementary mechanisms, including the following: i) alleviation dysbiosis of gut microbiota; ii) improvements in the intestinal barrier function; iii) decreased BT; and iv) reduced liver inflammatory response.

In response to liver injury, quiescent HSCs are activated and develop a myofibroblast-like phenotype that proliferates and expresses intermediate filament α-SMA and profibrotic genes^16^. We found that in the LI01 and LI05 groups, HSC activation was inhibited, the hepatic inflammatory response was decreased, and the expression of inflammatory mediators and profibrogenic cytokine genes, including TNF-α, TGF-β, INOS-2, IL-6 and IL-17A, were down regulated. Our results are consistent with other studies in which probiotics showed anti-inflammatory effects in experimentally induced cirrhosis^17, 18^. TGF-β is a major factor in the promotion of fibrogenesis in the liver and other tissues. Proinflammatory cytokines, such as IL-6, TNF-α and IL-17A, are key stimulators of HSC activation^19, 20^. The down-regulation of inflammatory cytokine expression may be related to lower endotoxin or bacterial DNA levels from the gut^21^. Furthermore, the down-regulation of INOS-2 gene expression indicated another potential mechanism mediating the anti-inflammatory effects of LI01 and LI05, which are likely related to their antioxidative inhibition of proinflammatory signalling in the chronically injured liver. Liver function is indicated by ALT, AST and GGT levels, which were reduced in the groups treated with the LI01 and LI05 strains, resulting in the alleviation of both hepatocyte damage and liver inflammation.

HSCs provide a link between the gut and the liver through their high expression of TLRs, which are pattern recognition receptors (PRRs) involved in sensing and eliminating antigens and thereby promote HSC activation and fibrosis^22^. The tissue sections with mild liver fibrosis in the LI01 and LI05 groups showed depressed TLR family gene expression. TLR2 promotes hepatic fibrosis-mediated injured intestinal permeability, leading to increased liver exposure to bacterial products from the gut microflora, which promote liver injury, inflammation, and fibrosis^23^. TLR4 plays a key role in the development of liver fibrosis; lipopolysaccharide (LPS) ligand from the gut microbiota activates TLR4 on HSCs, influencing downstream signalling and resulting in myofibroblast proliferation and ECM production^22^. TLR5 and its ligand bacterial flagellin are directly involved in the progression of fibrosis via activation of the NF-$\kappa$ B and MAPK signalling pathways^24^. TLR9, which is activated by bacterial DNA, is also required for liver fibrosis through the induction of IL-1β, which in turn activates the IL-1 receptor on HSCs, resulting in their activation^25^. Additionally, ECM is involved in hepatic fibrosis and is regulated by the specific inhibitor tissue inhibitor of metalloproteinases (TIMPs)^26^. The decreased expression of TIMP-1 in several probiotic strains suggested their beneficial effects on ECM regulation during fibrogenesis.

In the clinic, intestinal barrier function is impaired in patients with cirrhosis and parallels cirrhosis progression^27^. The improved hepatic fibrosis in the LI01 and LI05 groups was associated with their protective effect on the integrity and microstructure of the intestinal barrier and the resultant reduction in BT. This finding is similar to the results of previous studies showing that modulating the gut microbiota to correct intestinal barrier function and reduce BT and endotoxaemia could contribute to improving liver fibrogenesis^17, 28^. BT is considered to play a pivotal pathophysiological role in the development of severe complications, such as ascites, renal failure and hepatic encephalopathy^29^. The mechanism by which probiotics reduced BT can be simply summarized as gut flora homeostasis, which improves intestinal barrier functions and reduces inflammatory immune responses^18, 30^. Interestingly, in our present study, five probiotic strains increased the ileal expression of the antimicrobial peptide β-defensin-1. Antimicrobial peptides act as intestinal immune defences by maintaining intestinal barrier homeostasis^31^. The increased expression of β-defensin-1 may partially explain the decreased BT in the blood following treatment with *L. rhamnosus* GG or *C. butyricum*.

Multiple pathogenic factors accelerating liver injuries were associated with gastrointestinal microbiota dysbiosis. In this study, since we only sequenced partial 16 S rDNA sequences, and the OTUs could not be assigned to the species level with high confidence, none of the five probiotics strains and their genera were observed to be enriched in the caecal microbiome. This is likely because of no significant increases in the amount of these strains in the rat caecum 1 week after probiotic withdrawal. Importantly, the abundances of *Sutterellaceae* and *Escherichia*/*Shigella* (LPS-producing bacteria), *Erysipelotrichaceae* (inflammation-related bacteria), *Pasteurellaceae* and *Pseudoflavonifractor* (opportunistic pathogens) were highly enriched in the cirrhotic rats, whereas beneficial bacteria, such as *Akkermansia*, were decreased^32–35^. Probiotic interventions have been shown to effectively modulate gut barrier integrity and gut microbiota in animals by tempering chronic inflammation and metabolic disorders^35^. An increased F/B ratio, caused by an expansion of Firmicutes and/or a contraction of Bacteroidetes has been widely considered a signature of gut dysbiosis^36, 37^. The reduction of F/B ratio in LI01 or LI05 group demonstrated *L. salivarius* LI01 and *P. pentosaceus* LI05 produces beneficial effects on dysbiosis. Specifically, *L. salivarius* LI01 and *P. pentosaceus* LI05 alleviated microbial dysbiosis to promote intestine health, such as the depletion of *Escherichia*/*Shigella*, *Oscillospira*, *Oscillibacter*, and *Flavonifractor* and the enrichment of *Prevotella*, *Paraprevotella*, *Lactobacillus*, and *Elusimicrobium*. These gut bacteria that are specifically altered in probiotic LI01- and LI05-treated rats, especially enriched beneficial microbial taxa and inhibited opportunistic pathogens, may be involved in the amelioration of liver cirrhosis. Consistent with this conclusion, increases in liver injury indicators, profibrogenic genes, and inflammatory cytokines are negatively associated with enrichments of the beneficial taxa in probiotic LI01- and LI05-treated rats, such as the intensive association of *Prevotella* with IL-6 and TNF-α; *Elusimicrobium* with IL-6 and IL-17; *Lactobacillus* with GGT, Collagen α1 and TIMP-1. As reported, *Prevotella*, which was reported to be more abundant in healthy subjects than that in NAFLD patients, has been suggested to exert protective effects against the development of NAFLD^38^. *Elusimicrobium minutum* produces acetate and alanine, which may be beneficial for liver damage and the glucose–alanine cycle^39^. Symbiotic-containing *Lactobacillus paracasei* significantly reduced the expression of α-SMA, collagen, and inflammatory cytokines and improved liver inflammation and fibrosis in the same experimental rat model of liver cirrhosis^17^. However, the *Oscillibacter*-like organisms, including *Oscillibacter* and *Oscillospira*, were identified as potentially harmful gut microbes that mediated high saturated fat diet-induced gut dysfunction; additionally, consistent with our results, a negative correlation was found between the abundance of *Oscillibacter* and barrier function^40^. LPS is a major product from *Escherichia*/*Shigella*, and the overgrowth of these bacteria can impair intestinal permeability and result in endotoxaemia, which is associated with worsening disease severity and complications in cirrhosis^41^. Thus, the reduction in endotoxin level in probiotic LI01- or LI05-treated rats and the strong positive correlation between *Escherichia/Shigella* and TLRs suggested that the beneficial microbiome regulation of *L. salivarius* LI01 and *P. pentosaceus* LI05 ameliorated liver fibrosis. Additionally, gut microbial richness has been correlated with health^42^, and our previous study found a lower abundance of microbial diversity and richness in acute-on-chronic liver failure patients^43^. More speciose bacterial communities reduce pathogens more effectively. Thus, based on the Chao1 index and OTUs, the increase in microbial richness following long-term treatment with the specific probiotics LI01 and LI05 may play an important role in maintaining intestinal homeostasis.

In conclusion, the interaction of gut bacteria with different probiotic strains influences the progression of liver damage, which provides strong evidence that gut microbiome homeostasis contributes to the improvement of liver cirrhosis. The current study potentially provides new insights into the prevention and treatment of liver cirrhosis via selection of an appropriate probiotic strain to manipulate the gut microbiota.

## Electronic supplementary material

Supplementary materials
