# Supplementary material for: Administration of Lactobacillus salivarius LI01 or Pediococcus pentosaceus LI05 prevents CCl4-induced liver cirrhosis by protecting the intestinal barrier in rats
Source: Sci Rep. 2017 Jul 31;7:6927. doi: 10.1038/s41598-017-07091-1 (PMC5537250; doi:10.1038/s41598-017-07091-1)

Administration of *Lactobacillus salivarius* LI01 or *Pediococcus pentosaceus* LI05 prevents CCl_4-induced liver cirrhosis by protecting the intestinal barrier in rats

Ding Shi[a,1], Longxian Lv[a,1], Daiqiong Fang[a,1], Wenrui Wu[a,1], Chenxia Hu[a,1], Lichen Xu[a,1], Yanfei Chen[a], Jing Guo[a], Xinjun Hu[a], Ang Li[a], Feifei Guo[a], Jianzhong Ye[a], Yating Li[a], Dewi Andayani[a], Lanjuan Li[a*]

[a]State Key Laboratory for Diagnosis and Treatment of Infectious Diseases, Collaborative Innovation Centre for Diagnosis and Treatment of Infectious Diseases, the First Affiliated Hospital, College of Medicine, Zhejiang University, 310003 Hangzhou, China.

[1] Ding Shi, Longxian Lv, Daiqiong Fang, Wenrui Wu, Chenxia Hu, and Lichen Xu contributed equally to this work.

Supplementary materials

Supplementary Table. 1. PCR primer sets for RT-PCR analysis of the indicated genes.

Gene	Forward Sequence (5'-3')	Reverse Sequence (5'-3')	Product Length (bp)
β-defensin-1	GCTGCCCATCTCATACCAA	ACAATCCCTTGCTGTCCTTT	110
ZO-1	TGAGAAGCAGACACCCACTC	TTGGCAGAACACCATCAGAA	104
TGF-β1	ATTCCTGGCGTTACCTTGG	AGCCCTGTATTCCGTCTCCT	120
Timp-1	CTGGTTCCCTGGCATAATCT	ATCGCTCTGGTAGCCCTTCT	183
Col1a1	ATCTCCTGGTGCTGATGGAC	GCCTCTTTCTCCTCTCTGACC	109
TNF-α	CAGGTTCCGTCCCTCTCATA	TGCCAGTTCCACATCTCG	100
TLR4	CCCTGCCACCATTTACAGTT	ATCAGAGTCCCAGCCAGATG	100
TLR2	AAACTGTGTTCGTGCTTTCTG	GCGTCATTGTTCTCGTCAAA	100
TLR5	GGCTCAACCAAACCAATGTC	TCCTCTTCATCGCAGTCCTT	121
TLR9	GTCCCAGCCTTCTTTGCTCT	CGTCAGGTTCATCACAATGG	111
INOS-2	ACCTTTATGTTTGTGGCGATG	TCAACCTGCTCCTCACTCAA	100
IL-10	GAAGCTGAAGACCCTCTGGA	TGGCCTTGTAGACACCTTTG	131
IL-6	AGTTGCCTTCTTGGGACTGA	ACTGGTCTGTTGTGGGTGGT	102
IL-17a	GCGTCCTAAACAGAGACCTGA	AGGGTGGAAGGCAGACAAT	148

Supplementary Table. 2. Histological scores of the inflammatory cell, goblet cell, mucosa, villi architecture and crypts.

Group	Inflammatory Cell	Goblet cell	Mucosa	Crypts	Villi architecture	Total scores
Control	0.3 ± 0.2**	0.6 ± 0.2**	0.4 ± 0.2**	0 (0, 1.0)*	0 (0, 1.0)**	1.6 ± 0.3**
MY	2.4 ± 0.3	1.7 ± 0.2	1.6 ± 0.3	3.0 (0, 4.0)	3.0 (0, 3.0)	10.0 ± 1.3
ZCS	2.4 ± 0.3	2.1 ± 0.2	1.9 ± 0.1	1.5 (0, 3.75)	1.5 (0, 3.0)	9.6 ± 1.2
LI01	1.7 ± 0.2*	1.7 ± 0.2	1.8 ± 0.2	0 (0, 3.0)	0 (0, 3.0)*	7.8 ± 0.9
LI05	1.4 ± 0.3*	1.6 ± 0.2	1.7 ± 0.2	0 (0, 3.0)	0 (0, 3.0)*	7.0 ± 0.9*
LGG	2.4 ± 0.2	1.6 ± 0.3	2.9 ± 0.2	3.0 (0, 3.0)	1.5 (0, 3.0)	10.3 ± 0.9**
NS	2.6 ± 0.4	1.7 ± 0.3	2.1 ± 0.3	3.0 (0, 3.0)	3.0 (3.0, 3.0)	11.1 ± 0.9

All data are given as the mean ± SEM or median (25th, 75th percentiles). $^{*}P < 0.05$, $^{**}P < 0.01$ vs. normal saline-treated cirrhotic rats in the NS group.

Supplementary Table. 3. Comparison of diversity estimation of the 16S rRNA gene library at 97% similarity from the pyrosequencing analysis

Diversity index	Control	MY	ZCS	LI01	LI05	LGG	NS
OTUs	1918 ± 228	1969 ± 261	2223 ± 155**	2322 ± 200**	2197 ± 213**	1963 ± 204	1909 ± 183
Chao1	2956 ± 345	3237 ± 339	3679 ± 212**	3930 ± 273**	3695 ± 350**	3266 ± 288	3032 ± 280
Shannon	8.23 ± 0.30	8.17 ± 0.47	8.46 ± 0.41	8.40 ± 0.35	8.34 ± 0.46	8.24 ± 0.35	8.33 ± 0.17
Simpson	0.986 ± 0.005	0.980 ± 0.019	0.985 ± 0.001	0.985 ± 0.010	0.982 ± 0.002	0.987 ± 0.004	0.988 ± 0.003

All data are given as the mean ± SEM. $^{**}P < 0.01$ vs. normal saline-treated cirrhotic rats in the NS group. OTUs: operational taxonomic units.

Supplementary Table. 4. Clustering of the gut microbiota from different probiotic groups

Unifrac distance matrix used for ANOSIM	Group	Hypothesis	P-value	R-value
Unweighted	LI01	LI01 vs. Control	0.001^{**}	0.99
		LI01 vs. NS	0.001^{**}	0.80
		LI01 vs. MY	0.001^{**}	0.52
		LI01 vs. ZCS	0.002^{**}	0.42
		LI01 vs. LGG	0.001^{**}	0.67
		LI01 vs. LI05	0.002^{**}	0.33
Weighted	LI01	LI01 vs. Control	0.001^{**}	0.55
		LI01 vs. NS	0.001^{**}	0.48
		LI01 vs. MY	0.016^{*}	0.29
		LI01 vs. ZCS	0.001^{**}	0.45
		LI01 vs. LGG	0.001^{**}	0.43
		LI01 vs. LI05	0.008^{**}	0.21
Unweighted	LI05	LI05 vs. Control	0.001^{**}	0.86
		LI05 vs. NS	0.001^{**}	0.81
		LI05 vs. MY	0.008^{**}	0.30
		LI05 vs. ZCS	0.003^{**}	0.32
		LI05 vs. LGG	0.003^{**}	0.37
Weighted	LI05	LI05 vs. Control	0.001^{**}	0.31
		LI05 vs. NS	0.005^{**}	0.28
		LI05 vs. MY	0.543	-0.03
		LI05 vs. ZCS	0.05^{*}	0.17
		LI05 vs. LGG	0.139	0.08
Unweighted	NS	NS vs. Control	0.001^{**}	0.82
		NS vs. MY	0.002^{**}	0.62
		NS vs. LGG	0.002^{**}	0.32
		NS vs. ZCS	0.001^{**}	0.72
Weighted	NS	NS vs. Control	0.002^{**}	0.39
		NS vs. MY	0.095	0.127
		NS vs. LGG	0.319	0.02

		NS vs. ZCS	0.001**	0.63

Statistical tests between different probiotic groups based on weighted and unweighted UniFrac distance matrices. P-values < 0.05 are designated with *, <0.01 are designated with **. (ANOSIM with 999 permutations)

Supplementary Figure. 1. A PCoA plot of the microbiota based on the results of the weighted UniFrac metric. Each symbol represents a sample; the variance explained by the PCs is indicated on the axes.

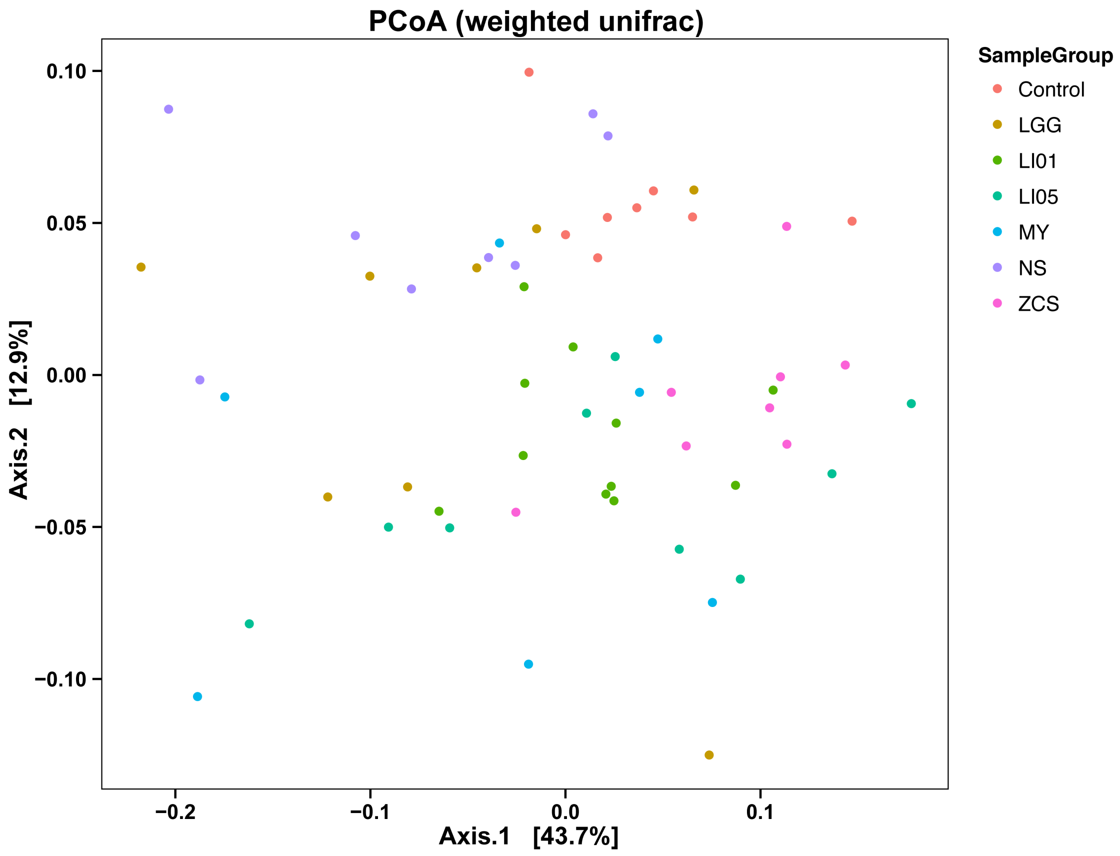

Supplement: Supplementary file 1 — Supplementary materials [file 41598_2017_7091_MOESM1_ESM.pdf]
